# Supplementary material for: Urinary Fluoride Levels Among Youth in the National Health and Nutrition Examination Survey (NHANES) 2015–2016: Potential Differences According to Race
Source: Nutrients. 2025 Jan 16;17(2):309. doi: 10.3390/nu17020309 (PMC11768995; doi:10.3390/nu17020309)
Supplement: Supplementary file 1 [file nutrients-17-00309-s001.zip › nutrients-3417275-supplementary.pdf]

Flow diagram

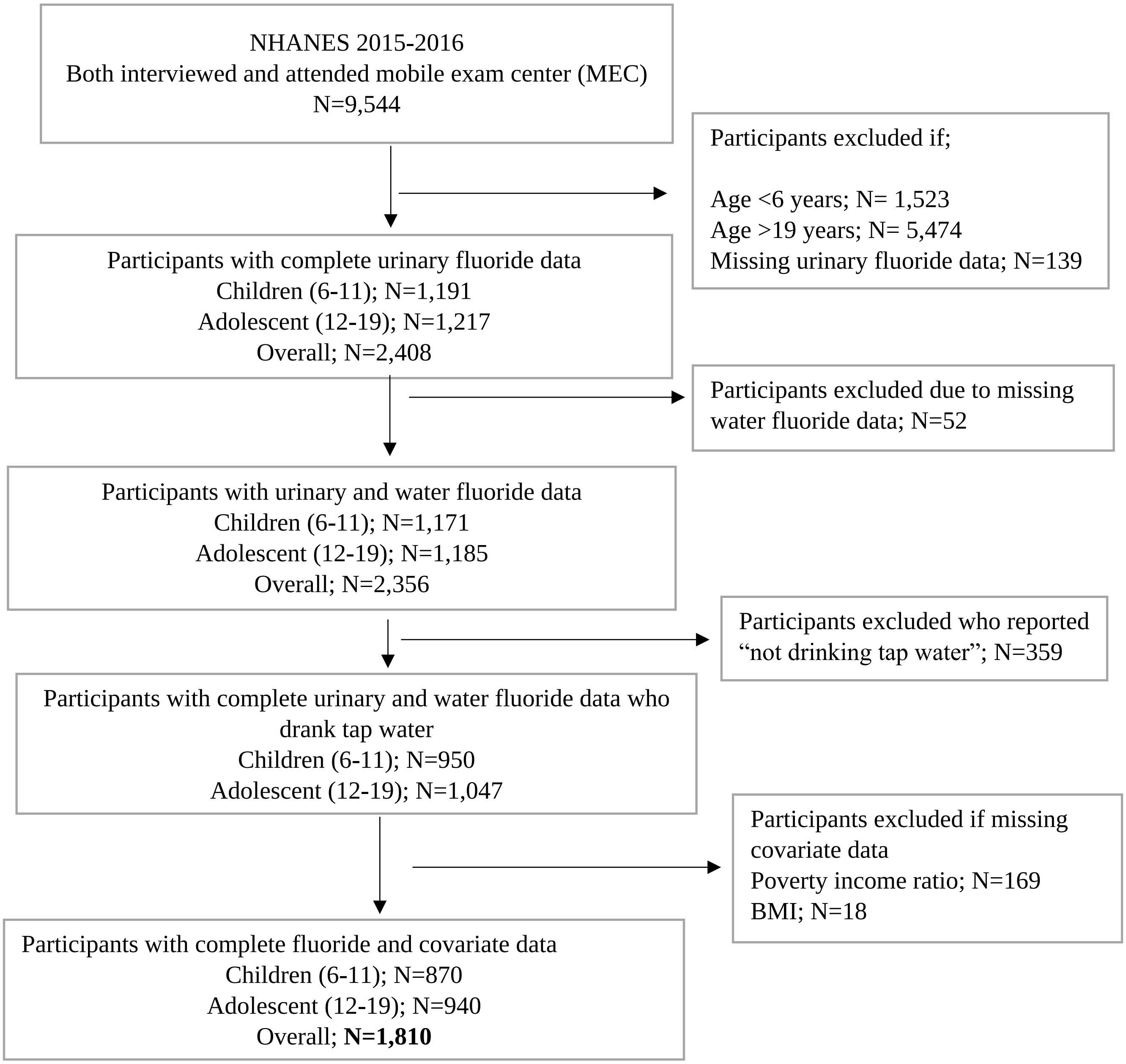

Supplementary Figure S1: NHANES study participant flowchart included in the final analysis

Table S1: Participants with complete data compared with all children/adolescents in NHANES 2015-2016		
	**Current study sample**	**Participants aged 6-19 years in NHANES 2015-2016**
**Total**<br>n=Unweighted<br>N=Weighted	 n=1,810[a]<br>N=44,575,958	 n=2,188[b]<br>N=52,320,980
**Age; Mean (SD)**	12.53 (3.80)	12.44 (3.83)
**Sex; Freq (%)**		
Male	23,316,522 (52.31)	26,924,436 (51.46)
Female	21,259,437 (47.69)	25,396,544 (48.54)
**Race/Ethnicity; Freq (%)[b]**		
Mexican American	5,803,702 (13.02)	7,079,364 (13.53)
Other Hispanic	3,458,436 (7.76)	4,221,273 (8.07)
Non-Hispanic White	25,533,062 (57.23)	2,890,8602(55.25)
Non-Hispanic Black	5,323,433 (11.94)	6,779,939 (12.96)
Non-Hispanic Asian	2,005,268 (4.50)	2,507,033 (4.79)
Other race/multi-racial	2,452,055 (5.50)	2,824,767 (5.40)
**BMI; Mean (SD)**	21.78 (5.96)	21.71(5.90)
**Ratio of family income to poverty; Mean (SD)**	2.58 (1.58)	2.60 (1.58)

[a] The final study sample with complete data of urinary, water fluoride, and covariates
[b] The study sample includes the participants with missing information on urinary, water fluoride and covariates (n=1810 plus n=378)
Reported frequencies are column percentages
All the estimates, mean, SD, frequencies (%) were calculated using NHANES survey MEC weights

Table S2: Creatinine-adjusted urinary fluoride (UF_{CR}) levels among different age groups			
	**Median[a] (IQR)**	**Mean[a] (SD)**	**5th, 95th Percentiles**
**Children (6-11 years)**			
**UF_{CR} (mg/L)[b]**<br>N = 22,809,623 (unweighted n =1,191)	0.64 (0.44)	0.78 (0.77)	0.28,1.69
**Adolescent (12-19 years)**			
**UF_{CR} (mg/L)**<br>N = 32,237,630 (unweighted n =1,217)	0.56 (0.44)	0.68 (0.48)	0.25,1.38
**Overall Sample (6-19 years)**			
**UF_{CR} (mg/L)**<br>N = 55,047,254 (unweighted n = 2,408)	0.60 (0.50)	0.74 (0.74)	0.23,1.66

Creatinine-adjusted urine fluoride = UF_{CR}; IQR, Inter Quartile Range; SD, Standard Deviation
Estimates were calculated using NHANES survey MEC weights
UF_{CR} concentrations were calculated for each age-group sub-sample separately (children, adolescents and overall) by dividing the urinary fluoride concentration in the sample by the sample's creatinine concentration and multiplying the product by the average creatinine concentration for the sample.

Table S3: Weighted urinary fluoride levels across different sociodemographic factors among youth										
	Children (6-11 years)					Adolescents (12-19 years)				
**Socio-demographic factors**	N	Mean(SD)	Median (IQR)	Min	Max	N	Mean(SD)	Median (IQR)	Min	Max
**Sex**										
Male	11,768,499	0.74 (0.51)	0.65 (0.56)	0.10	4.45	16,610,112	0.63 (0.45)	0.52 (0.46)	0.10	3.02
Female	11,041,124	0.61 (0.56)	0.47 (0.46)	0.10	10.99	15,627,518	0.55 (0.42)	0.45 (0.48)	0.10	3.1
**Race/Ethnicity**										
Mexican American	3,714,328	0.68 (0.73)	0.52 (0.54)	0.10	10.99	4,708,357	0.61 (0.50)	0.45 (0.46)	0.10	2.99
Other Hispanic	2,298,391	0.60 (0.38)	0.52 (0.44)	0.10	1.88	2,678,689	0.56 (0.43)	0.43 (0.52)	0.10	2.48
Non-Hispanic White	11,268,033	0.65 (0.47)	0.56 (0.54)	0.10	4.45	17,267,764	0.57 (0.42)	0.48 (0.44)	0.10	3.1
Non-Hispanic Black	3,031,464	0.87 (0.64)	0.70 (0.70)	0.10	4.48	4,495,378	0.70 (0.46)	0.57 (0.54)	0.10	3.02
Non-Hispanic Asian	1,164,985	0.57 (0.47)	0.41 (0.47)	0.10	2.88	1,492,885	0.46 (0.40)	0.34 (0.44)	0.10	2.55
Other race/multi-racial	1,332,420	0.62 (0.45)	0.54 (0.49)	0.10	2.61	1,594,554	0.67 (0.44)	0.61 (0.52)	0.10	2.52
**Body Mass Index (BMI)**										
Underweight	564,147	0.63 (0.56)	0.48 (0.51)	0.10	2.88	1,210,014	0.63 (0.42)	0.54 (0.34)	0.15	1.99
Normal Weight	14,498,796	0.65 (0.44)	0.55 (0.56)	0.10	4.39	17,744,547	0.58 (0.42)	0.48 (0.48)	0.10	3.02
Overweight	3,637,561	0.71 (0.55)	0.59 (0.51)	0.10	4.48	6,072,413	0.60 (0.46)	0.47 (0.52)	0.10	2.96
Obese	40,62,406	0.74 (0.79)	0.58 (0.46)	0.10	10.99	6,715,593	0.62 (0.50)	0.51 (0.47)	0.10	3.1
Missing	46,711	0.39 (0.13)	0.31 (0.22)	0.26	0.52	495,061	0.62 (0.36)	0.62 (0.60)	0.10	1.66
All estimates were calculated using NHANES survey MEC weights										

Table S4: Proportion of participants of each race/ethnicity, sex, and BMI category, below, at, or above the previously recommended tap water fluoride level range			
**Children (6-11 years) N=950**			
	**Less than 0.7 mg/L %[a]**	**0.7 - 1.2 mg/L % [a]**	**Above 1.2 mg/L % [a]**
**Race/Ethnicity**			
Mexican American N=206	70.39	15.53	14.08
Other Hispanic N=122	86.07	9.84	4.10
Non-Hispanic White N=282	76.95	20.92	2.13
Non-Hispanic Black N=191	59.16	40.84	0.00
Non-Hispanic Asian N=81	77.78	22.22	0.00
Other Race/Multi-Racial N=68	70.59	29.41	0.00
**Sex**			
Male N=478	69.46	25.52	5.02
Female N=472	76.06	20.55	3.39
**Body Mass Index (BMI)**			
Underweight N=24	75.0	25.0	0.00
Normal Weight N=578	72.32	24.57	3.11
Overweight N=157	80.25	15.29	4.46
Obese N=187	66.84	25.13	4.23
Missing N=6	--	--	--

Adolescents (12-19 years) N=1,047			
	**Less than 0.7 mg/L % [a]**	**0.7 - 1.2 mg/L % [a]**	**Above 1.2 mg/L % [a]**
**Race/Ethnicity**			
Mexican American N=215	71.63	20.47	7.91
Other Hispanic N=119	77.31	20.17	2.52
Non-Hispanic White N=303	82.51	16.17	1.32
Non-Hispanic Black N=246	63.41	36.59	0.00
Non-Hispanic Asian N=102	89.22	10.78	0.00
Other Race/Multi-Racial N=62	83.87	14.52	1.61
**Sex**			
Male N=549	77.23	20.22	2.55
Female N=498	74.50	23.29	2.21
**Body Mass Index (BMI)**			
Underweight N=29	72.41	27.59	0.00
Normal Weight N=571	76.36	20.84	2.80
Overweight N=199	76.38	21.61	2.01
Obese N=227	75.33	22.47	2.20
Missing N=21	--	--	--

[a] Reported frequencies are row percentages and unweighted
Categories based on the recommended fluoride level of 0.7-1.2mg/L prior to the change in recommendation in 2015

Table S5: Covariate-adjusted linear regression for associations between log-transformed water fluoride and urinary fluoride			
	**Unweighted n (weighted N)**	**β (95% CI)**	***p*-value**
**Children (6-11 years)**			
**Urine Fluoride (mg/L)**	870 (17,903,881)	0.14 (0.11, 0.16)	<0.001
**Adolescent (12-19 years)**			
**Urine Fluoride (mg/L)**	940 (26,672,077)	0.13 (0.10, 0.15)	<0.001
**Overall (6-19 years)**			
**Urine Fluoride (mg/L)**	1,810 (44,575,958)	0.13 (0.11, 0.15)	<0.001

Water fluoride and urinary fluoride were natural log transformed.
Participants who reported that they did not drink the tap water were excluded; β Coefficients and 95% CIs are rescaled according to an IQR (ie, 0.54mg/L for children; 0.53 mg/L for adolescents; 0.54 for overall) increase in water fluoride level. The β estimates, 95% CIs and *p*-values were calculated using NHANES survey MEC weights. MEC weights were re-weighted to the dietary sample for regression analyses. All models are adjusted for age, sex, race/ethnicity, BMI, ratio of family income to poverty, and urine creatinine levels; unweighted samples sizes are n = 870 for children, n = 940 for adolescents, n = 1,810 for the overall sample

Table S6: Relative associations between water fluoride and urinary fluoride according to race/ethnicity with non-Hispanic White as the reference

	**25th Quantile** β estimates (95% CI)	**50th Quantile** β estimates (95% CI)	**75th Quantile** β estimates (95% CI)
**Children 6-19 years; N =17,903,881 (unweighted n= 870)**			
WF X Mexican American	-0.04 (-0.10, 0.03)	-0.02 (-0.08, 0.04)	0.03 (-0.08, 0.14)
WF X Other Hispanic	0.01 (-0.05, 0.07)	-0.03 (-0.13, 0.08)	0.01 (-0.19, 0.21)
WF X Non-Hispanic White	Reference	Reference	Reference
WF X Non-Hispanic Black	0.13 (0.01, 0.25)*****	0.12 (0.01, 0.24)*****	0.16 (0.03, 0.30)*
WF X Non-Hispanic Asian	-0.04 (-0.23,0.15)	-0.06 (-0.25, 0.13)	-0.15 (-0.50, 0.19)
WF X Other race/multi-racial	-0.002 (-0.16, 0.15)	0.05 (-0.10, 0.20)	0.13 (-0.16, 0.41)
**Adolescent 12-19 years; N=26,672,077 (unweighted n=940)**			
WF X Mexican American	-0.04 (-0.09,0.01)	0.005 (-0.07, 0.08)	0.07 (-0.05, 0.20)
WF X Other Hispanic	-0.03 (-0.12, 0.06)	-0.05 (-0.18, 0.09)	0.04 (-0.18, 0.26)
WF X Non-Hispanic White	Reference	Reference	Reference
WF X Non-Hispanic Black	0.01 (-0.07, 0.09)	0.06 (-0.06, 0.19)	0.09 (-0.11, 0.28)
WF X Non-Hispanic Asian	-0.07 (-0.22, 0.08)	-0.05 (-0.29, 0.18)	0.04 (-0.33, 0.41)
WF X Other race/multi-racial	0.05 (-0.07, 0.17)	0.08 (-0.11, 0.27)	0.09 (-0.21, 0.39)
**Overall 6-19 years; N=44,575,958 (unweighted n= 1,810)**			
WF X Mexican American	-0.04 (-0.08, -0.002)*****	0.01 (-0.05, 0.07)	0.05 (-0.02, 0.13)
WF X Other Hispanic	-0.02 (-0.09, 0.05)	-0.03 (-0.13, 0.08)	-0.03 (-0.16, 0.10)
WF X Non-Hispanic White	Reference	Reference	Reference
WF X Non-Hispanic Black	0.07 (-0.001, 0.14)[a]	0.13 (0.03, 0.23)*****	0.18 (0.05, 0.31)*****
WF X Non-Hispanic Asian	-0.03 (-0.15, 0.10)	-0.04 (-0.22, 0.14)	0.002 (-0.22, 0.23)
WF X Other race/multi-racial	0.002 (-0.10, 0.10)	-0.024 (-0.17, 0.12)	0.13 (-0.05, 0.31)

Associations were examined using survey-weighted and covariate-adjusted quantile regression;
β estimates and 95% CI are rescaled to an IQR (ie, 0.54mg/L for children; 0.53 mg/L for adolescents; 0.54 for overall) increase in water fluoride levels. The β estimates, 95% CIs and *p*-values were calculated using NHANES survey MEC weights. MEC weights were re-weighted to the dietary sample for regression analyses; Participants who reported that they did not drink the tap water were excluded
All models are adjusted for age, sex, race/ethnicity, BMI, ratio of family income to poverty, and urine creatinine levels
*****p-value <0.05
[a] marginal p=0.052

**Table S7: Covariate-adjusted linear regression of relative associations between log-transformed water fluoride and urinary fluoride according to race/ethnicity with non-Hispanic white as the reference**		
	**β (95% CI)**	***p*-value**
**Children (6-11 years)** 870 (17,903,881)		
**Urine Fluoride (mg/L)**		
WF X Mexican American	0.03 (-0.03, 0.09)	0.31
WF X Other Hispanic	-0.01 (-0.08, 0.06)	0.81
WF X Non-Hispanic White	Reference	Reference
WF X Non-Hispanic Black	0.07 (0.004, 0.13) *	0.04*
WF X Non-Hispanic Asian	-0.05 (-0.17, 0.07)	0.42
WF X Other race/multi-racial	0.01 (-0.07, 0.09)	0.82
**Adolescent (12-19 years)** 940 (26,672,077)		
**Urine Fluoride (mg/L)**		
WF X Mexican American	0.05 (-0.01,0.11)	0.09
WF X Other Hispanic	0.02 (-0.05, 0.09)	0.53
WF X Non-Hispanic White	Reference	Reference
WF X Non-Hispanic Black	0.01 (-0.04, 0.07)	0.62
WF X Non-Hispanic Asian	0.01 (-0.06, 0.09)	0.67
WF X Other race/multi-racial	0.03 (-0.08, 0.13)	0.60
**Overall (6-19 years)** 1,810 (44,575,958)		
**Urine Fluoride (mg/L)**		
WF X Mexican American	0.04 (-0.01, 0.09)	0.08
WF X Other Hispanic	0.0003 (-0.05, 0.05)	0.99
WF X Non-Hispanic White	Reference	Reference
WF X Non-Hispanic Black	0.04 (-0.01, 0.08)	0.08
WF X Non-Hispanic Asian	-0.01 (-0.08, 0.06)	0.75
WF X Other race/multi-racial	0.01 (-0.06, 0.07)	0.88

Water fluoride (WF) and urinary fluoride were natural log-transformed .
Participants who reported that they did not drink the tap water were excluded; β Coefficients and 95% CIs are rescaled according to an IQR (ie, 0.54mg/L for children; 0.53 mg/L for adolescents; 0.54 for overall) increase in water fluoride levels. The β estimates, 95% CIs and *p*-values were calculated using NHANES survey MEC weights. MEC weights were re-weighted to the dietary sample for regression analyses.

All models are adjusted for age, sex, race/ethnicity, BMI, ratio of family income to poverty, and urine creatinine levels; unweighted samples sizes are n = 870 for children, n = 940 for adolescents, n = 1,810 for the overall sample
*****p-value <0.05

**Table S8: Ratio of family income to poverty according to race/ethnicity among youth**												
**Children (6-11 years)**							**Adolescents (12-19 years)**					
**Race/Ethnicity**	N	Mean (SD)	Median (IQR)	Min	Max	*p*-value	N	Mean (SD)	Median (IQR)	Min	Max	*p*-value
						<0.001						<0.001
Mexican American	187	1.55 (1.23)	1.23 (1.43)	0	5		182	1.46 (1.19)	1.23 (1.17)	0	5	
Other Hispanic	105	1.86 (1.34)	1.47 (1.82)	0	5		107	1.84 (1.30)	1.49 (1.63)	0.2	5	
Non-Hispanic White	271	2.74 (1.49)	2.47 (2.58)	0	5		295	2.76 (1.50)	2.5 (2.63)	0	5	
Non-Hispanic Black	174	1.34 (1.22)	0.95 (1.33)	0	5		204	1.56 (1.24)	1.23 (1.4)	0	5	
Non-Hispanic Asian	72	3.06 (1.65)	3.06 (3.64)	0	5		93	2.78 (1.60)	2.49 (2.84)	0	5	
Other race/multi-racial	61	2.79 (1.65)	2.46 (2.89)	0	5		59	2.25 (1.51)	2.06 (2.24)	0	5	
Note. **n=1810;** the estimates are unweighted; reported *p*-values were calculated using unweighted non-parametric Kruskal–Wallis tests												
